# Complete Laparoscopic Excision of a Primary Isolated Mesenteric Hydatid Cyst With Radiological Features Suggestive of Inactivity

**DOI:** 10.7759/cureus.117625

**Published:** 2026-10-08

**Authors:** Simon Gilliard, Francisco Da Rocha De Sousa, Fabrice Corbisier

**Affiliations:** 1 General Surgery, Cliniques Universitaires Saint-Luc, Brussels, BEL; 2 General Surgery, Grand Hôpital de Charleroi, Charleroi, BEL

**Keywords:** abdominal mass, case report, cystic echinococcosis, inactive cyst, laparoscopy, mesenteric hydatid cyst

## Abstract

Primary isolated mesenteric hydatid cysts are uncommon, and management is uncertain when imaging suggests an inactive lesion. We describe a 25-year-old patient in whom an abdominal mass was incidentally identified during a routine examination. An initial abdominopelvic computed tomography scan revealed an approximately 8-cm supravesical cystic lesion suspicious for a degenerating hydatid cyst. Magnetic resonance imaging showed a thickened, calcified wall, complex internal fluid, and no abnormal enhancement. Cerebral and thoracoabdominal imaging disclosed no other suspected hydatid focus; *Echinococcus* serology was negative. After specialist multidisciplinary assessment, surgery was selected because of lesion size, its unusual supravesical location, and concern about possible compression of adjacent structures, despite imaging features suggestive of inactivity. The cyst was excised laparoscopically in one piece without intra-abdominal rupture or bowel resection, and was removed in a retrieval bag. Histopathology was compatible with a remodeled, calcified/ossified hydatid cyst wall, although *Echinococcus* polymerase chain reaction did not detect *Echinococcus* DNA. No perioperative albendazole was administered. Recovery was uncomplicated, discharge occurred on postoperative day 2, and the patient remained clinically well at three months. This report illustrates a carefully selected laparoscopic approach while emphasizing that evidence supporting surveillance of inactive hepatic cysts cannot be directly generalized to isolated mesenteric lesions.

## Introduction

Cystic echinococcosis is a zoonotic parasitic infection caused by the larval stage of *Echinococcus granulosus* sensu lato. Humans acquire infection through ingestion of parasite eggs shed by infected canids. Hydatid cysts most frequently develop in the liver and lungs, although virtually any tissue can be involved [1]. The mesentery, the peritoneal fold that supports the intestine and contains its vascular supply, is an uncommon site of disease.

Primary isolated mesenteric involvement is rare and has largely been described in individual case reports [2-5]. In a 2026 retrospective series of 523 patients who underwent surgery for hydatid disease, only one case had a primary mesenteric location; the study was not designed to establish treatment indications for inactive mesenteric cysts [6].

The World Health Organization Informal Working Group on Echinococcosis (WHO-IWGE) classification distinguishes active (CE1-CE2), transitional (CE3), and inactive (CE4-CE5) cyst morphology and was originally based on ultrasound [7,8]. Current WHO guidance mainly addresses uncomplicated hepatic cysts and does not establish a dedicated management algorithm for apparently inactive mesenteric lesions [1]. We therefore report a presumed primary isolated mesenteric hydatid cyst with radiological features suggestive of inactivity, removed completely by laparoscopy without intra-abdominal rupture, and discuss the resulting diagnostic and therapeutic uncertainty.

## Case presentation

A 25-year-old patient living in Belgium, with no relevant medical or surgical history, was referred after an abdominal mass was incidentally detected during a routine medical examination. The patient reported no abdominal pain or gastrointestinal symptoms. The abdomen was soft and nontender, and deep palpation identified a firm subumbilical mass that was mobile relative to the abdominal wall and deeper planes. The patient reported no known relevant animal exposure. A remote one-month stay in Africa during childhood was noted; however, neither the source, location, nor timing of a possible infection could be established.

An initial abdominopelvic computed tomography (CT) scan, performed in early July 2025 to characterize the mass, showed an approximately 8-cm supravesical cystic lesion considered suspicious for a degenerating hydatid cyst. The patient was referred to an infectious diseases service for further investigation.

Pelvic magnetic resonance imaging (MRI) performed in July 2025 demonstrated an 8 × 6.8 × 5.2-cm supravesical mass with a thickened wall, mural calcifications, complex thick-appearing fluid, and internal projections. No abnormal enhancement was described after gadolinium administration. The radiological impression favored a degenerating, apparently inactive hydatid cyst. The relevant CT, T2-weighted MRI, and post-gadolinium MRI appearances are shown in Figure 1.

**Figure 1:**
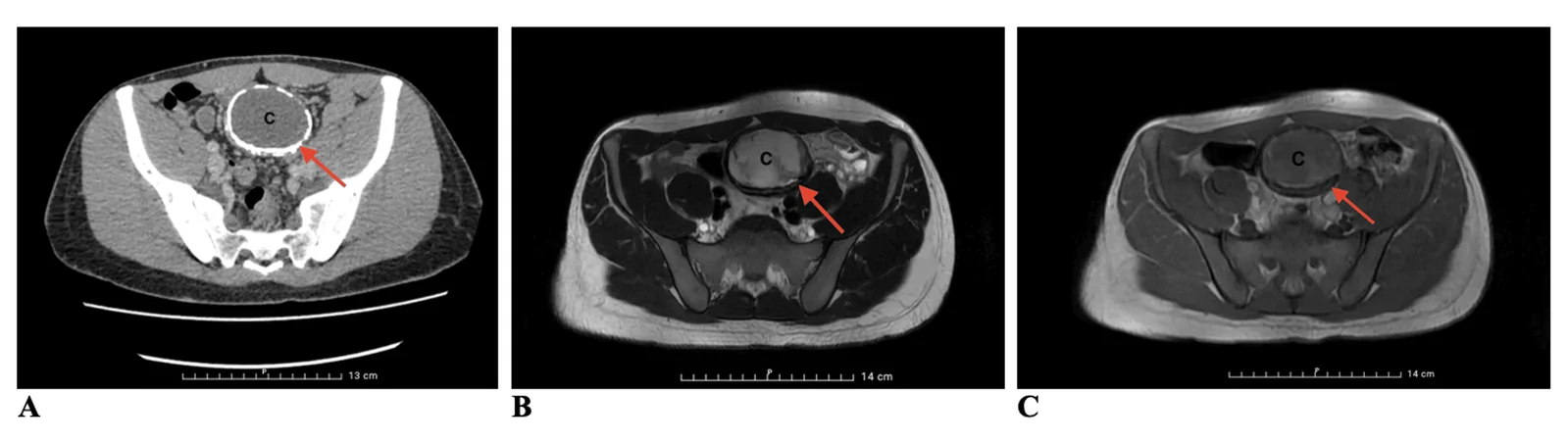
Preoperative imaging. (A) Portal venous-phase axial CT showing a well-defined supravesical cystic lesion with prominent, nearly circumferential mural calcification (arrow). (B) Axial T2-weighted MRI showing a thick-walled lesion with heterogeneous internal signal (arrow). (C) Post-gadolinium axial T1-weighted MRI showing no appreciable abnormal enhancement of the cyst wall (arrow). The letter C in the source images denotes the cyst. CT: computed tomography; MRI: magnetic resonance imaging.

Subsequent cerebral and thoracoabdominal CT imaging in August 2025 demonstrated the known supravesical lesion, measuring approximately 7 × 8 cm, without any additional suspected cerebral, thoracic, or abdominal hydatid focus. Preoperative imaging localized the lesion to the supravesical region but did not definitively establish its organ of origin. Its mesenteric origin was confirmed during laparoscopic exploration. Serology for *E. granulosus* immunoglobulin G and *E. multilocularis* EM2/EM18 antibodies was negative.

Following assessment by infectious diseases specialists, the patient was referred to the surgical team. The case was discussed with a specialized echinococcosis group at a tertiary referral center. Although the images suggested degeneration or inactivity, the approximately 8-cm size, unusual intraperitoneal and supravesical location, and concern about potential mass effect on neighboring structures contributed to the decision to remove the lesion. No perioperative albendazole was advised by the specialist team.

Elective laparoscopy was performed in September 2025. A four-port technique was used, with an 11-mm port in the left hypochondrium, two 5-mm ports in the left hypochondrium and left flank, and an 11-mm port in the right iliac fossa. Exploration revealed a large, firm cyst arising within the mesentery. Gauze pads soaked in hypertonic saline were placed around the cyst as a peripheral scolicidal precaution in the event of inadvertent rupture. Dissection was performed close to the cyst wall with an energy-sealing device while preserving as much mesenteric tissue as possible. The cyst was removed completely in one piece without intra-abdominal perforation, and the resulting mesenteric defect was closed with a running suture. The intact specimen was placed in a retrieval bag and removed through an approximately 7-cm suprapubic Pfannenstiel incision. After reinsufflation, the small bowel appeared healthy and well perfused, and bowel resection was unnecessary (Figure 2). Estimated blood loss was minimal.

**Figure 2:**
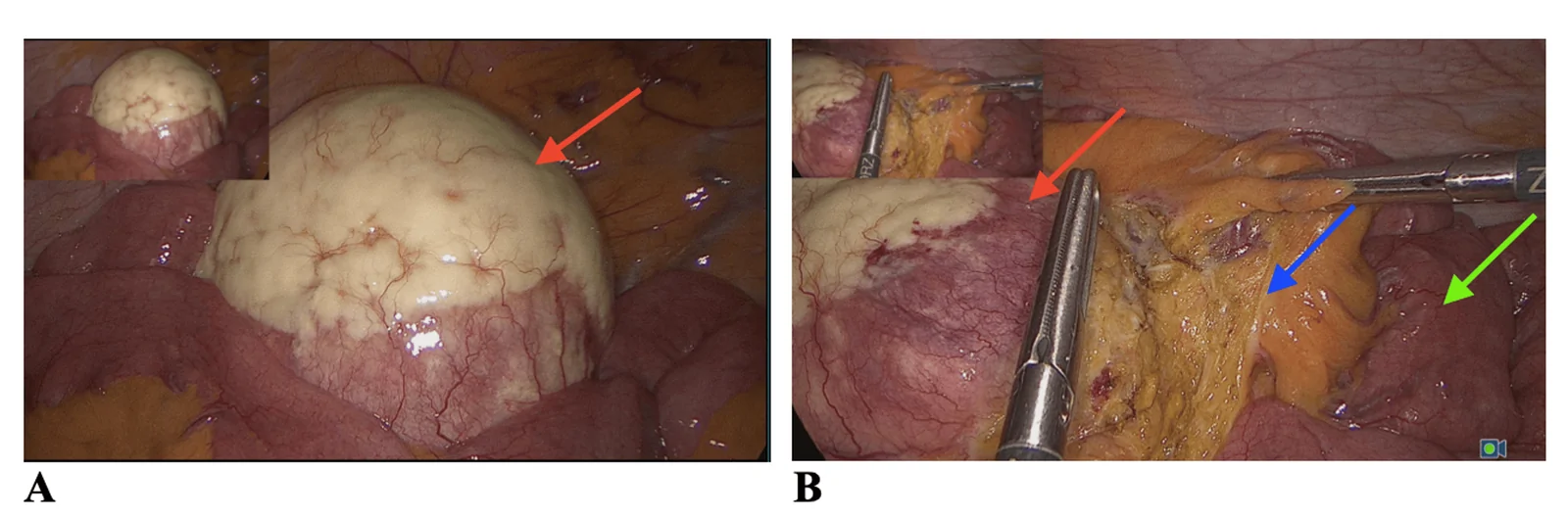
Intraoperative laparoscopic views. (A) Large cystic lesion identified within the mesenteric region during laparoscopic exploration (red arrow). (B) Careful dissection along the cyst wall with preservation of surrounding mesenteric tissue, showing the cyst (red arrow), adjacent mesenteric tissue being divided (blue arrow), and the neighboring small bowel loop (green arrow). The small inset images in the corners are automatically generated duplicate thumbnails from the laparoscopic capture system and do not represent additional operative views.

The pathology specimen measured 11 × 7 × 6 cm. These ex vivo specimen dimensions were larger than the approximately 8-cm maximum diameter measured on preoperative imaging. This discrepancy may reflect differences in measurement plane and the fact that pathology recorded the dimensions of the entire resection specimen rather than an in vivo radiological cyst diameter. After protected extracorporeal retrieval, it was opened outside the abdominal cavity, and the cyst contained turbid brownish fluid without distinctive macroscopic features. No daughter cysts or detached parasitic membranes were reported (Figure 3).

**Figure 3:**
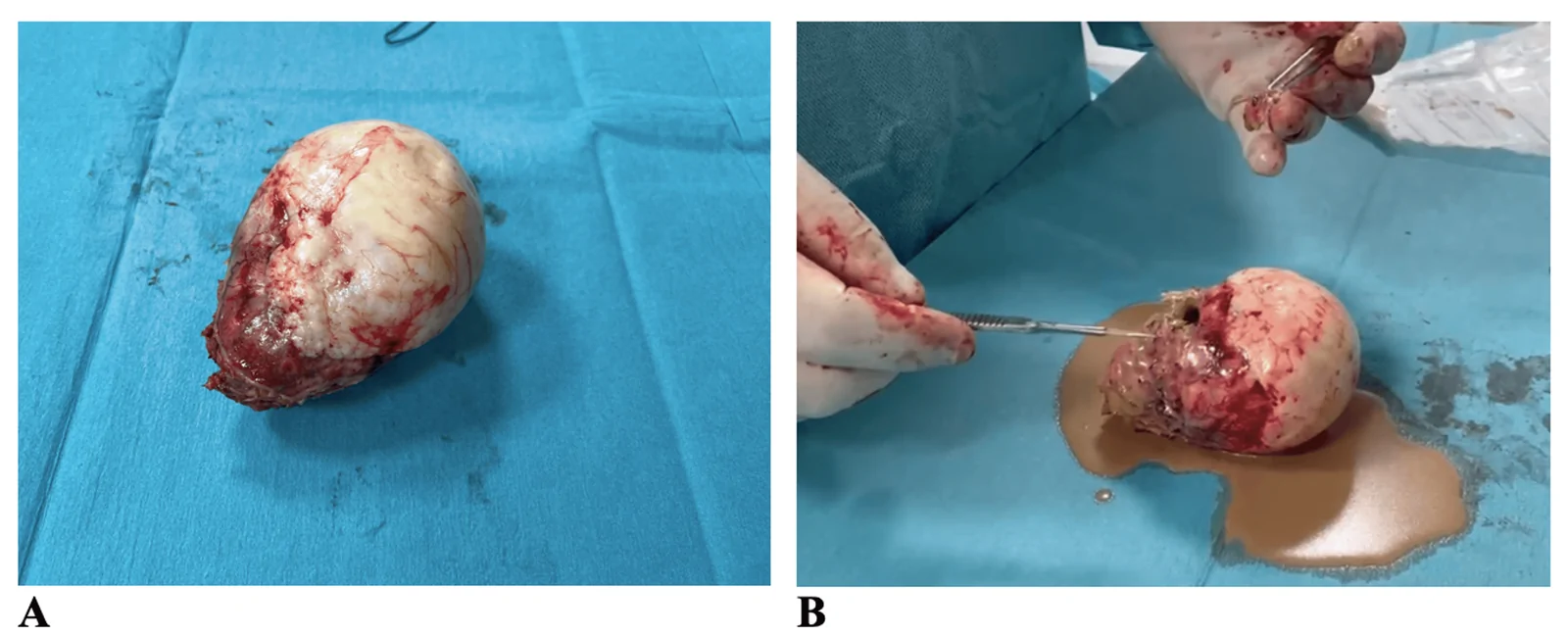
Gross appearance of the excised mesenteric lesion after extracorporeal retrieval. (A) Intact specimen before opening. (B) The specimen was opened only after its removal from the abdominal cavity; liquid material is visible adjacent to the opened cyst.

The specimen required prolonged decalcification; the final histopathology report, issued in October 2025, described a remodeled, calcified/ossified cyst wall compatible with a hydatid cyst, without suspicious malignant features. *Echinococcus* polymerase chain reaction (PCR), reported in September 2025, detected no *Echinococcus* DNA. The available laboratory documentation did not specify the molecular target or whether the material tested had undergone decalcification. No parasite-specific structures were reported, and parasite viability could not be established.

The postoperative course was uncomplicated, and the patient was discharged on postoperative day two. At the one-month surgical follow-up, the patient reported no abdominal, gastrointestinal, or other complaints, and abdominal examination was unremarkable. At the three-month clinical review, the patient remained asymptomatic and in good general condition. Further follow-up will be conducted by the infectious diseases team.

## Discussion

Isolated mesenteric location and diagnostic certainty

Primary isolated mesenteric involvement is uncommon; secondary intra-abdominal involvement may follow spontaneous or iatrogenic rupture of a cyst elsewhere. In this patient, the absence of previous hydatid disease or abdominal surgery, the negative extension workup, and direct operative identification of an intramesenteric lesion support the classification as isolated primary mesenteric disease, although the route of infection cannot be established [2-6].

Diagnosis of cystic echinococcosis is primarily based on imaging, with serology providing supplementary evidence. Negative serology does not exclude cystic echinococcosis [1]. In a retrospective imaging study, Stojkovic et al. found higher agreement with ultrasound-based staging for MRI than for CT in CE1-CE4 cysts (κ = 0.83-1.00 vs. 0.62-0.72), whereas CT showed higher agreement for CE5 (κ = 0.95 vs. 0.65), primarily because of its superior depiction of wall calcifications [9]. In our patient, the initial CT findings and MRI interpretation suggested a degenerating hydatid cyst. The histopathological findings were compatible but not parasite-specific, and both serology and *Echinococcus* PCR were negative. The diagnosis therefore rests on convergent radiological and histopathological evidence rather than definitive species identification or parasitological confirmation.

Radiological inactivity and the limits of staging

The WHO-IWGE classification was developed from ultrasound. Its morphological categories can be applied beyond the liver, except in osseous disease, but stage-dependent treatment recommendations cannot automatically be transferred to other anatomical sites [1,7,8]. CE4 and CE5 are classified as inactive; a typical CE5 cyst has a completely or almost completely eggshell-calcified wall and degenerative solid internal material [1].

Our patient had no dedicated staging ultrasound. Although CT demonstrated marked, almost circumferential calcification and MRI suggested degeneration, the available reports did not establish all CE5 characteristics. They described residual complex fluid, which was also seen when the resected specimen was opened. Neither the presence of calcification nor the lack of abnormal enhancement alone establishes nonviability. Accordingly, we describe imaging features suggestive of inactivity without assigning a definitive WHO-IWGE stage. Calcification can be present in active or transitional lesions; Hosch et al. demonstrated this across different cyst stages [10]. Najih et al. similarly described a calcified primary mesenteric cyst that still contained daughter cysts [2].

Treatment decision: Surveillance or surgery?

For uncomplicated inactive hepatic cysts, current WHO guidance describes imaging follow-up, often termed a watch-and-wait approach, as standard practice and discourages surgery unless complications arise [1]. In a cohort of 47 previously untreated inactive hepatic cysts, Piccoli et al. observed one reactivation during a median follow-up of approximately 52 months [11]. In the prospective cohort of Stojkovic et al., none of the 46 cysts that had become inactive without treatment reactivated over a mean follow-up of 5.4 years, whereas eight of 17 cysts rendered inactive after benzimidazole therapy reactivated [12]. A subsequent scoping review reinforces this distinction between spontaneously and treatment-induced inactivity [13]. These results arise predominantly from hepatic disease and cannot establish the natural history of a mesenteric lesion.

In our patient, lesion size alone was not treated as a stand-alone indication. Rather, the approximately 8-cm diameter, unusual supravesical intraperitoneal location, concern about potential compression, and feasibility of complete removal informed a multidisciplinary decision. No compression was objectively documented, and the available evidence does not demonstrate that surgery was superior to surveillance for this particular lesion. The present observation should not be interpreted as an argument for routine excision of all apparently inactive mesenteric cysts.

The absence of perioperative albendazole was an individualized recommendation from a specialized echinococcosis group at a tertiary referral center, made in the context of imaging features suggestive of inactivity, rather than an application of a mesenteric-specific WHO recommendation. WHO recommendations combining surgery and albendazole address defined hepatic cyst scenarios and do not provide a corresponding algorithm for an apparently inactive mesenteric cyst [1]. A contemporary retrospective series of atypical primary locations reported postoperative albendazole use, but it did not evaluate inactive mesenteric lesions separately or compare treatment strategies [6]. In our case, complete excision was achieved without intra-abdominal rupture or visible spillage.

Role of laparoscopy

Open surgery remains the predominant approach reported for mesenteric hydatid cysts. In a review of heterogeneous uncommon intra-abdominal sites by Mihetiu et al., an open approach was used in 85.71% of the analyzed cases; the small sample and mixed disease patterns limit the generalizability of this figure [14].

Laparoscopy has nonetheless been reported previously for primary mesenteric disease by Yagmur et al. and, more recently, for a CE2 mesenteric lesion by Chandankhede et al. [3,5]. Mihetiu et al. also reported laparoscopic resection of mesenteric lesions in multifocal abdominal hydatidosis [14].

Here, close dissection enabled intact extracorporeal retrieval, conservation of mesenteric vascular supply, and avoidance of bowel resection. The hypertonic saline pads were a peripheral precaution only; no intracystic scolicidal agent was instilled. The specimen was opened only after removal from the abdominal cavity. This experience demonstrates technical feasibility in one selected patient, but provides no comparative evidence of superiority over laparotomy.

Limitations

The absence of dedicated staging ultrasound and incomplete documentation of the defining CE5 morphology preclude confident stage assignment. Moreover, histopathology was compatible with, rather than definitive for, hydatid disease, and both serology and *Echinococcus* PCR were negative. The liquid content observed after extracorporeal opening does not establish parasite viability but reinforces the need for cautious terminology. Postoperative follow-up is currently limited to three months and has been clinical only. This single observation cannot establish the natural history of an untreated mesenteric lesion or compare treatment strategies.

## Conclusions

An isolated mesenteric cyst with radiological features suggestive of inactivity presents a management question for which stage-specific evidence is scarce. In this patient, size, anatomical location, and concern about potential mass effect prompted an individualized decision for surgery despite imaging findings suggesting degeneration.

Complete laparoscopic excision without intra-abdominal rupture or bowel resection was feasible. The case illustrates a technical option for carefully selected lesions, while underscoring that surveillance evidence derived predominantly from inactive hepatic cysts cannot be directly generalized to mesenteric disease. Further reports are needed to better define patient selection and the role of laparoscopy in this uncommon setting.
